# Divergent risk patterns across coastal hospitality sectors in the Northern Adriatic

**DOI:** 10.12688/openreseurope.23489.1

**Published:** 2026-06-03

**Authors:** Vilane G. Sales

**Affiliations:** 1 Department of Environmental Sciences Informatics and Statistics, Ca' Foscari University of Venice, Venice, Veneto, Italy; 2 Leibniz Centre for Tropical Marine Research (ZMT) GmbH, Bremen, Bremen, Germany; 3 Foundation Euro-Mediterranean Center for Climate Change, Venezia, Veneto, Italy

**Keywords:** coastal vulnerability, hospitality risk index, H3 hexagonal indexing, fuzzy clustering, spatial autocorrelation, Northern Adriatic, platform accommodation, IPCC risk framework

## Abstract

Coastal hospitality, encompassing both accommodation (hotels, holiday rentals, short-term rental platforms) and service establishments (restaurants, bars, tourism-oriented retail and leisure), along the Northern Adriatic coast exhibits fundamentally different spatial risk patterns across sectors. This study demonstrates this divergence by developing a dual-component Hospitality Risk Index (HRI) applied to 10,022 establishments across 400 km of coastline in Veneto and Emilia-Romagna, combined with fuzzy clustering and H3 hexagonal spatial indexing. The HRI integrates hazard projections (sea-level rise under IPCC scenarios), exposure metrics (elevation, coastal proximity, spatial density), and vulnerability indicators (structural characteristics, economic positioning). Accommodation risk differentiates by hazard geography: converted residential properties concentrated in the Venice lagoon system (HRI = 0.422) score substantially higher than purpose-built coastal hotels (HRI = 0.231), a pattern driven by differential hazard exposure that survives removal of the vulnerability component. Service risk follows a simpler spatial logic, with beachfront establishments showing highest scores (HRI = 0.440) regardless of economic positioning. These sectoral differences manifest as geographically concentrated hotspots, with service risk exhibiting two- to four-fold stronger spatial clustering than accommodation risk. External validation against two coastal flood events (Venice 2019, Emilia-Romagna 2022) confirms that flood-proximate establishments score significantly higher (p < 0.001), with the difference driven by the exposure component. The framework provides a spatial baseline for monitoring vulnerability configurations over time and offers a transferable open-source methodology for comparable Mediterranean coastal destinations.

## 1. Introduction

Italy ranks among the European countries with highest natural disaster probability, with 65% of its population residing in medium-to-high hazard zones ( Pagliacci and Russo 2020), and socioeconomic exposure to flooding continues to increase as development concentrates in hazard-prone areas ( Amadio et al. 2019). Coastal regions in Northern Italy, particularly along the Adriatic Sea, host a hospitality sector generating approximately €20 billion annually in tourism revenue within this hazard landscape ( Demoskopika 2023). This sector encompasses accommodation (hotels, holiday rentals, platform-mediated short-term rentals) and service (restaurants, bars, tourism-oriented retail and leisure) industries. This infrastructure faces increasing exposure to sea-level rise, coastal erosion, and intensified storm surges ( Perini et al. 2017; Galassi and Spada 2014; Anzidei et al. 2020), and the majority of establishments positioned within 2 km of the shoreline ( Anzidei et al. 2020; Reimann et al. 2018).

Yet despite this economic significance and hazard exposure, a basic geographic question remains unanswered: do accommodation and service establishments, which locate according to fundamentally different spatial logics, exhibit different spatial risk patterns? Hotels sort along amenity-hazard gradients from the coastline inland; restaurants and bars cluster by agglomeration in commercial districts; converted residential properties occupy locations selected for residential amenity rather than hazard avoidance. No study has compared these sectors at establishment level, an omission that matters because undifferentiated destination-level assessments obscure the within-sector heterogeneity that targeted adaptation requires ( Bahja et al. 2024; Soontiens-Olsen et al. 2023). The rapid expansion of platform-mediated short-term rentals in converted residential buildings adds urgency: their spatial distribution and risk characteristics relative to purpose-built hotels remain undocumented in this region ( Gutiérrez et al. 2017; Wachsmuth and Weisler 2018).

This study asks three questions. First, how do spatial risk patterns differ between accommodation and service establishments along the Northern Adriatic coast? Second, what hospitality typologies emerge from fuzzy clustering across amenity, market, and structural dimensions, and how do these typologies relate to risk scores? Third, do risk distributions cluster geographically, and do clustering patterns differ between sectors? To answer these questions, the study develops a dual-component Hospitality Risk Index (HRI) applied to 10,022 establishments, identifies typologies through Fuzzy C-means clustering, and characterizes spatial organization through exploratory spatial data analysis (ESDA) using H3 hexagonal indexing (~0.5 km ^2^).

The analysis reveals that the two sectors diverge sharply: accommodation risk differentiates by hazard geography, with converted residential properties in the Venice lagoon scoring highest, while service risk follows a simpler spatial logic driven by direct coastal proximity. The design is explicitly cross-sectional and descriptive. Spatial patterns are documented and interpreted through established theoretical frameworks, without claiming causal mechanisms.

## 2. Literature review

### 2.1. Vulnerability, place, and coastal tourism development

Vulnerability is produced through socio-spatial processes that concentrate risk in particular locations and among particular populations ( Wisner et al. 2004). The hazards-of-place tradition established that vulnerability arises from the intersection of biophysical risk exposure and social context at specific sites ( Cutter 1996; Cutter et al. 2003), a principle extended to coupled human-environment systems where development decisions reshape the hazard landscapes to which communities are exposed ( Turner et al. 2003). Through successive IPCC assessment reports ( IPCC 2007, 2014), this spatial logic was formalized as the risk-as-interaction framework: risk arises from the interaction of hazard, exposure, and vulnerability, where vulnerability subsumes both sensitivity and adaptive capacity ( Bevacqua et al. 2018). This study adopts that tripartite architecture. Existing coastal vulnerability indices, from Gornitz’s (1990) foundational Coastal Vulnerability Index to more recent multi-hazard applications ( Kunte et al. 2014), typically treat the built environment as a static exposure surface rather than as the material outcome of historical development decisions, a limitation that building-level assessment can begin to address. The hazard event does not cause vulnerability; it reveals pre-existing conditions produced by development decisions ( Calgaro et al. 2014).

This place-based logic applies with particular force to coastal tourism destinations. Butler’s (1980) tourism area life cycle shows how destinations produce characteristic infrastructure during each development phase, infrastructure that persists through subsequent market cycles. Agarwal’s (2002) restructuring thesis supplies the critical mechanism: the built environment created during growth phases outlasts the economic logic that produced it. This persistence creates “path plasticity” ( Clavé and Wilson 2017), whereby development trajectories are substantially constrained by inherited built form. Demolition and reconstruction costs exceed adaptive reuse, and sunk investment discourages relocation even as hazard conditions deteriorate. Crucially, component sectors within a resort (accommodation, attractions, services) react differently to restructuring pressures ( Agarwal 2002, 2005), producing sector-specific vulnerability configurations rather than uniform destination-level risk. Resort morphology research confirms this lock-in: the spatial structure of tourism areas becomes fixed through early land-use decisions, with beachfront parcels allocated to high-amenity uses and secondary locations receiving lower-intensity development ( Liu and Wall 2009; Yang et al. 2018). The amenity value that attracts hospitality investment (beach proximity, waterfront access, scenic settings) coincides spatially with the hazard characteristics that produce flood exposure; building age, height, footprint, and proximity to shoreline all encode decades of development decisions that simultaneously configured economic value and hazard exposure.

Beyond exposure, adaptive capacity modifies the resulting vulnerability configuration. Adaptive capacity is primarily a function of economic resources ( IPCC 2014; Smit and Wandel 2006; Mortreux and Barnett 2017). For hospitality establishments, price tier serves as a proxy for the economic resources available for structural reinforcement, insurance, and post-event recovery; establishments commanding higher prices presumably generate greater revenue for risk mitigation investment ( Adger 2003). This operationalization introduces a tension: price tier reflects both adaptive capacity and location quality, while location quality correlates with coastal proximity, and therefore hazard exposure. The same beachfront premium that generates revenue for risk mitigation simultaneously signals higher exposure. Disentangling these overlapping effects requires component-level analysis that separates hazard-driven from vulnerability-driven risk variation. How these overlapping effects manifest spatially depends on the location logics that govern where different types of hospitality establishments locate.

### 2.2. Hospitality location, platform restructuring, and sectoral vulnerability

Accommodation and service establishments locate according to distinct spatial logics, with different implications for hazard exposure and vulnerability. Hotels locate according to a spatial hierarchy dominated by amenity proximity, accessibility, and bid-rent sorting ( Yang et al. 2018; Valenzuela-Ortiz et al. 2025). Beach and waterfront proximity serves as the primary determinant of coastal hotel siting, generating a distance-decay gradient from the shoreline in which higher-category hotels occupy beachfront positions and lower-cost establishments locate progressively inland ( Marco-Lajara et al. 2016; Gelbman 2021). This bid-rent gradient creates a systematic spatial sorting of hazard exposure. The establishments with the greatest amenity-driven revenue occupy the locations with the greatest coastal hazard exposure, while budget establishments are sorted into inland positions with lower exposure but also lower revenue-generating capacity. Star-rating stratification thus produces a spatial pattern where economic resources for adaptation, proxied by price following established adaptive capacity operationalizations ( Adger 2003; Smit and Wandel 2006), and hazard exposure are positively correlated, complicating the standard assumption that higher adaptive capacity reduces net vulnerability. Hotel location logic is oriented around amenity value rather than hazard avoidance, a spatial regularity that directly connects hotel siting decisions to the place-based vulnerability framework outlined above.

By contrast, service establishments (restaurants, bars, retail and leisure) follow a distinct spatial logic with different implications for hazard exposure. Where hotels locate primarily by amenity proximity to natural features, services locate by foot traffic, agglomeration economies, and co-location with accommodation clusters ( Tian et al. 2023; Chhetri et al. 2013). Hotels generate captive demand that attracts restaurant clustering in their vicinity; dining and entertainment options in turn enhance accommodation attractiveness, creating mutual spatial attraction with distinct spatial signatures for each sector. The key distinction is the mechanism driving clustering: hotels cluster along coastlines because each establishment independently seeks shoreline proximity, while services cluster because they seek proximity to each other and to the customer flows that hotels generate. Services therefore exhibit stronger spatial autocorrelation than accommodations because agglomeration economies (the benefits of proximity to complementary businesses and shared customer flows) dominate their location decisions more than direct amenity access ( Kim et al. 2021). These different location logics predict different hazard exposure profiles for the two sectors: hotels sort along an amenity-to-hazard gradient from coastline inland, while services cluster in commercial agglomerations whose coastal proximity depends on the broader destination structure rather than individual establishment siting preferences. In both cases, the amenity value driving coastal hospitality investment coincides spatially with the hazard characteristics producing flood exposure ( Student et al. 2020; Kellens et al. 2012).

A third category of establishments complicates this two-sector geography. Platform-mediated short-term rentals convert residential buildings into tourism accommodation, concentrating in historic centers, waterfront districts, and areas with building typologies that appeal to tourists seeking authentic experience ( Gutiérrez et al. 2017; Cerezo-Medina et al. 2022; Gyódi 2023). As tourist cores saturate, this conversion spreads to adjacent residential neighborhoods ( Hidalgo et al. 2023; Robertson et al. 2022; Wachsmuth and Weisler 2018). Converted residential properties differ systematically from purpose-built hotels in structural characteristics relevant to hazard resilience: they tend to be older, lower in height, built to residential rather than commercial construction standards, and located according to residential rather than commercial siting criteria ( Cerezo-Medina et al. 2022). Whether this restructuring concentrates vulnerability by placing tourism activity in buildings not designed for commercial use, in locations selected for residential amenity rather than hazard avoidance, is the question that connects platform accommodation geography to spatial risk assessment. Platform-mediated accommodation further reshapes the broader hospitality geography by displacing resident-serving businesses with tourist-oriented establishments ( Kim et al. 2021), a contingency that produces spatial heterogeneity motivating sectoral comparison.

### 2.3. From theory to measurement: analytical framework

Despite the spatial dynamics described above, three gaps persist in hospitality vulnerability research. First, spatial dimensions of hospitality vulnerability remain largely unexamined, with only 12% of coastal vulnerability studies achieving sub-municipal resolution ( Soontiens-Olsen et al. 2023), gap echoed across the broader coastal vulnerability mapping literature ( de Sherbinin et al. 2020). Second, services receive almost no vulnerability analysis distinct from hotels, despite following fundamentally different location logics ( Bahja et al. 2024). Third, the physical vulnerability of hospitality infrastructure itself remains poorly characterized. Tourism-climate research concentrates on demand-side impacts ( Demiroglu and Hall 2020), while existing destination-level frameworks ( Calgaro and Lloyd 2008; Espiner and Becken 2014; Becken et al. 2014; Santos-Lacueva et al. 2017) assess institutional and socioeconomic dimensions of tourism vulnerability but do not incorporate building-level structural characteristics (height, footprint, construction type) that determine how individual establishments withstand flooding, erosion, and sea-level rise.

If the vulnerability-producing processes described above operate through spatially structured mechanisms, vulnerability itself should exhibit spatial autocorrelation. Three mechanisms produce this expectation. First, shared hazard exposure: establishments in the same coastal zone face similar sea-level rise projections, storm surge patterns, and subsidence conditions. Second, shared development trajectory: neighboring establishments reflect the same historical investment wave, creating correlated building characteristics and vulnerability profiles ( Pagliacci and Russo 2020). Third, agglomeration effects: hospitality clustering creates correlated adaptive capacity within spatial clusters ( Hoover and Tate 2025). These mechanisms correspond to the three IPCC components, suggesting that spatial autocorrelation in a composite vulnerability index reflects genuine underlying spatial processes rather than methodological artifact. Assuming spatial stationarity masks critical localized patterns ( Hoover and Tate 2025), while construction choices in weighting and aggregation can amplify or suppress spatial patterns in ways that obscure the underlying geography of risk ( Reckien 2018; McLaughlin and Cooper 2010).

The theoretical architecture developed above generates four descriptive expectations that frame the analysis. First, establishments with direct beach access should show elevated risk regardless of market positioning, because exposure is a function of location, not price. Second, accommodations and services should exhibit different spatial risk signatures: accommodations sorted along an amenity-hazard gradient, services clustered by agglomeration in patterns that may or may not coincide with highest-hazard locations. Third, converted residential properties should show higher vulnerability than purpose-built hotels, because conversion selects for amenity-rich locations that overlap with hazard exposure. Fourth, budget establishments should show higher vulnerability independent of coastal proximity, if adaptive capacity is economically structured. These are framed as interpretive expectations guiding the descriptive analysis, not testable hypotheses.

The Northern Adriatic provides a setting where these mechanisms operate under contrasting physical conditions (Venice’s lagoonal system versus Emilia-Romagna’s linear Riviera) within a single national regulatory context; the study area is detailed in Section 3.1. The analytical design computes risk scores at establishment level, then aggregates to H3 hexagonal cells (~0.5 km ^2^) for spatial analysis, bridging the scale gap between individual-facility resolution and neighborhood-level spatial patterns.

## 3. Methodology

### 3.1. Study area

The study encompasses coastal Veneto and Emilia-Romagna in Northern Italy, regions with distinct geomorphological and tourism development profiles. Selecting these two regions provides a natural comparative design: Veneto’s coastline is dominated by the Venice Lagoon system and Po Delta, creating complex lagoonal environments where flood pathways are multi-directional, tidal dynamics interact with river discharge, and subsidence compounds sea-level rise. Emilia-Romagna, by contrast, presents a linear sandy coastline with relatively uniform exposure gradients. This geomorphological contrast allows examination of whether spatial risk patterns differ across fundamentally different coastal configurations, a question that single-region studies cannot address.

Veneto extends along the northern Adriatic, encompassing sandy beaches, shallow nearshore waters, and the Venice Lagoon system. Beach ridges and lagoonal deposits generated through fluvial inputs and nearshore hydrodynamics define the geomorphological structure ( Da Col et al. 2024). Extensive anthropogenic modifications have altered sediment transport patterns, particularly in zones of concentrated hospitality infrastructure ( Bonaldo et al. 2014; Ruol et al. 2018). The Venice Lagoon exemplifies complex anthropogenic-natural system interactions, with documented susceptibility to relative sea-level rise from subsidence and regional sea-level dynamics ( Zanchettin et al. 2021).

Emilia-Romagna extends approximately 130 km, exhibiting low-lying plains, reclaimed wetlands, and sandy beaches. The coastal economy depends predominantly on sun-sea-sand tourism, with 93 km (71%) of coastline extensively urbanized ( Armaroli et al. 2019). Historical subsidence combined with low elevations creates conditions conducive to seawater inundation during storm surge events ( Giambastiani et al. 2017; Perini et al. 2017).

**Figure 1.**
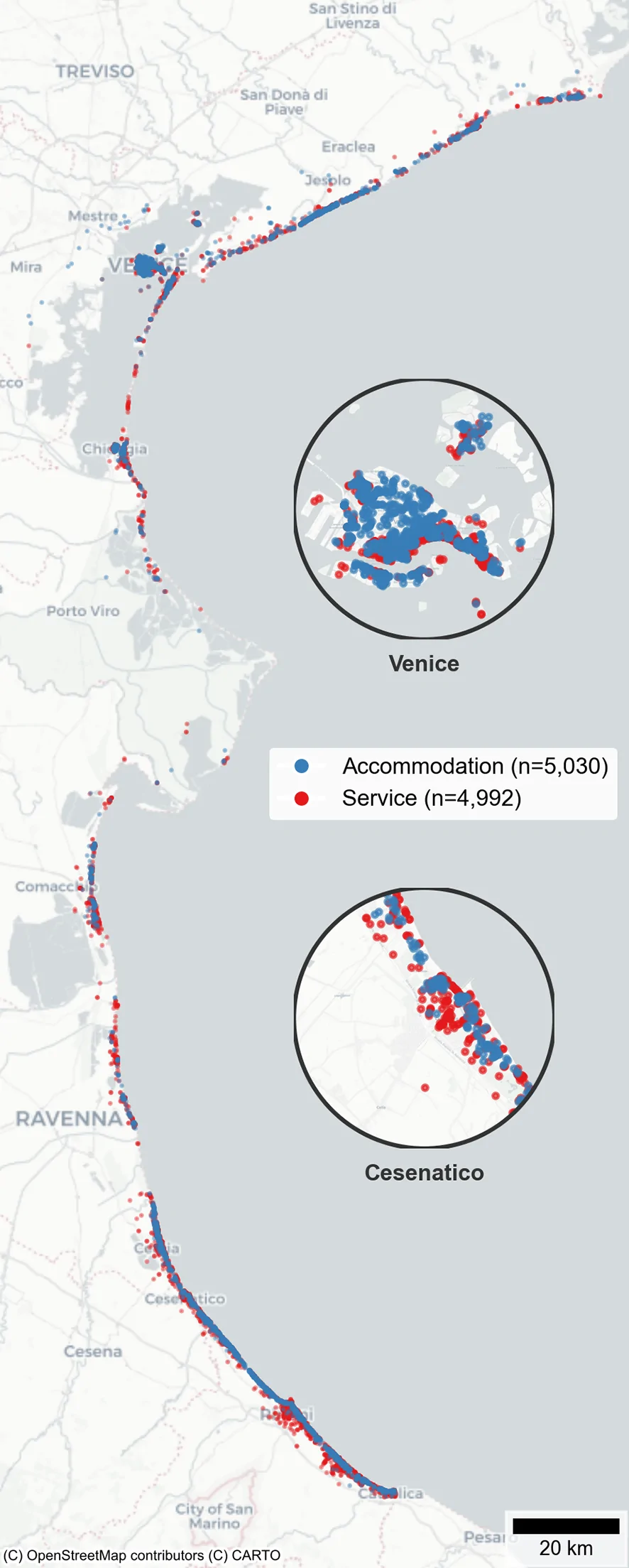
Distribution of 10,022 hospitality establishments across coastal Veneto and Emilia-Romagna, Northern Adriatic. Points differentiated by sector (accommodation, service).

The tourism development histories also diverge. Emilia-Romagna’s Riviera Romagnola developed through coordinated post-war investment in purpose-built hotel infrastructure and organized beach concession systems. Veneto’s hospitality landscape evolved more heterogeneously, with Venice’s historic urban fabric increasingly converted to platform-mediated short-term accommodation. These differences (lagoonal complexity versus linear beach development, residential conversion versus purpose-built corridors) provide variation for examining whether spatial risk patterns differ across coastal contexts ( Figure 1).

### 3.2. Data sources and variable construction

The hospitality database comprises 5,030 accommodation establishments and 4,992 service facilities. Accommodations include hotels, holiday rentals, bed-and-breakfasts, agritourism properties, and platform-mediated short-term rentals. Services include restaurants, bars, tourism-oriented retail, and leisure establishments. Variables are organized into Hazard, Exposure, and Vulnerability components following the IPCC (2014) risk assessment framework, in which vulnerability encompasses both sensitivity to harm and adaptive capacity to cope with or recover from impacts. This tripartite structure follows the operationalization approach demonstrated by Sharma and Ravindranath (2019) for hazard-specific vulnerability assessment.

Hazard variables quantify projected sea-level rise by combining IPCC-AR5 ensemble projections under RCP 4.5 scenarios for 2030 ( Church et al. 2013a, 2013b) with elevation data from TINITALY DEM ( Tarquini et al. 2023). Specifically, the difference between projected sea-level rise and establishment elevation is computed, with threshold-based scoring capturing non-linear risk escalation; establishments near or below projected sea levels receive disproportionately higher hazard scores. This approach serves as a proxy for inundation risk rather than a full hydrodynamic flood model.

Exposure variables include coordinates derived from Google Places API, Eubucco ( Milojevic-Dupont et al. 2023), Overture Maps Foundation (2024), and OpenStreetMap (2024); elevation above mean sea level; Euclidean distances to coastal features; and establishment clustering metrics through H3 hexagonal spatial indexing. Exposure integrates these physical parameters with spatial density measures, capturing both the position of individual establishments and the concentration of hospitality infrastructure in hazard-proximate areas.

Vulnerability variables integrate structural characteristics (building footprint, height, establishment type) with economic indicators (operational capacity, pricing, service quality ratings) and environmental features (beach access, including *stabilimenti balneari* or Italian beach concessions, and certifications). Service quality ratings capture the professional management dimension of adaptive capacity, complementing price as an economic proxy: establishments with higher ratings reflect more professionalized operations and presumably greater institutional capacity for risk mitigation. Following the adaptive capacity approach, establishments with greater economic resources and professional management infrastructure receive lower vulnerability scores, reflecting assumed greater capacity for risk mitigation investment. This operationalization means that the vulnerability component partially reflects business model type. Missing values are coded systematically (−1 for categorical, NA for numerical variables). Detailed data collection methodologies and validation protocols are documented in Sales (2024).

### 3.3. Hospitality risk index

The HRI synthesizes hazard, exposure, and vulnerability through a dual-component formula computed at establishment level:

$$\mathrm{HRI}=\alpha \times \left(\text{Hazard}\times \text{Exposure}\times \text{Vulnerability}\right)+\beta \times \left(a_{H}\times \text{Hazard}+a_{E}\times \text{Exposure}+a_{V}\times \text{Vulnerability}\right)\;$$ (1)

The dual-component formulation follows established multi-risk assessment practice ( Cotti et al. 2022; Chang et al. 2023; Lari et al. 2009; Marzocchi et al. 2012). The multiplicative component captures compounded effects when all dimensions are simultaneously elevated. The additive component preserves independent contributions, maintaining baseline risk signals when one or more components are moderate.

A caveat warrants emphasis: because the vulnerability component incorporates economic and operational characteristics, HRI scores partially reflect business model type. Establishments with fewer rooms, lower prices, and less professional infrastructure receive higher vulnerability scores by construction.

Methodological robustness is assessed through sensitivity analysis across 25 constrained parameter combinations. Component weights satisfy α + β = 1 (multiplicative-additive balance) and aH + aE + aV = 1 (additive sub-component balance), ensuring each combination represents a proportionally valid HRI formulation. The grid varies α from 0.3 to 0.7 across five additive weight vectors (default, hazard-dominant, exposure-dominant, vulnerability-dominant, and equal weighting). Coefficient of variation serves as the primary stability metric.

To assess whether the HRI captures genuine flood risk rather than methodological artifacts, establishment-level HRI scores are validated against observed flood extents from two Copernicus Emergency Management Service (EMS) activations ( Copernicus EMS 2019, 2022): EMSR409 (Venice coastal flood, November 2019; 570 flood delineation polygons) and EMSR642 (Emilia-Romagna compound coastal-riverine flood, November 2022; Comacchio, 6 polygons; Ravenna, 71 polygons). While EMSR642 involved both storm surge and riverine flooding, the flood extents overlap with the coastal hazard zone the HRI is designed to assess.

Establishment-level HRI is computed using the same formula as above but without H3 aggregation, preserving individual-establishment resolution. Spatial density, which is inherently an area-level measure, remains computed at H3 resolution and joined back to establishments. Each establishment is classified as flood-proximate if located within 50 m of a Copernicus-delineated flood polygon, or not flood-proximate otherwise. The 50 m buffer accounts for satellite-derived delineation uncertainty and ground-level water spread beyond mapped boundaries. To avoid comparing flood-affected establishments against the entire study area (which would conflate validation with a coastwide exposure test), the comparison group is restricted to establishments within the Copernicus Areas of Interest (AOI), the monitored zones where both flooded and non-flooded status can be reliably determined.

One-sided Mann-Whitney U tests ( Mann and Whitney 1947) assess whether flood-proximate establishments exhibit higher HRI than non-proximate establishments within the same monitored areas. This non-parametric test is chosen because HRI distributions are bounded, potentially skewed, and the comparison involves unequal group sizes, conditions under which parametric alternatives (t-tests) may yield unreliable inference. Rank-biserial correlation (r; Kerby 2014) provides a non-parametric effect size measure interpretable as the proportion of favorable pairs. Component-level comparisons (hazard, exposure, vulnerability separately) identify which HRI dimensions drive any observed differences.

### 3.4. Spatial framework and fuzzy clustering

The framework employs Uber’s H3 hierarchical spatial indexing system at resolution 7, corresponding to hexagonal cells of approximately 0.5 km ^2^. This resolution balances granular spatial pattern capture against statistical stability for aggregation. H3 provides three methodological advantages over rectangular grids. First, uniform spatial relationships and consistent neighbor distances across the study area. Second, hierarchical structure enabling seamless scale transitions. Third, optimal area coverage with minimal perimeter length, reducing boundary effects in coastal environments where land-water interfaces create complex geometric configurations. Establishment-level HRI scores and data are assigned to hexagonal cells, with additional operations computing dominant clusters (most prevalent cluster per cell) and proportional representation (cluster point counts relative to cell totals).

Within this spatial framework, the Fuzzy C-means (FCM) algorithm ( Dunn 1973; Bezdek 1981) identifies hospitality typologies based on variable combinations spanning IPCC framework components. Fuzzy approaches have been applied in spatial risk assessment contexts where crisp classification boundaries are inappropriate, including coastal hazard zonation ( Jadidi et al. 2014) and multi-parameter aquifer vulnerability mapping ( Javadi et al. 2022). Three clustering scenarios examine different analytical dimensions for each sector, each designed to capture a distinct mechanism from the theoretical framework. Amenity clustering integrates adaptive capacity proxies (pricing, service quality ratings) with environmental service provision (beach access, *balneari*, and certifications), operationalizing the economic dimension of vulnerability. Market clustering combines exposure variables with economic vulnerability indicators and structural characteristics, capturing the bid-rent sorting and coastal positioning mechanisms outlined in Section 2.2. Structural clustering focuses on physical building characteristics (height, footprint) integrated with coastal exposure measures and operational capacity, capturing infrastructure-based typologies whose physical characteristics may reflect different development origins. The clustering procedure minimizes the objective function:

$$J_{m}\left(U,V;X\right)=\sum_{i=1}^{c}\sum_{j=1}^{n}{\left(u_{\mathrm{ij}}\right)}^{m}d^{2}\left(X_{j},V_{i}\right)$$ (2)

subject to membership constraints ensuring each establishment’s memberships sum to unity across clusters. Validation employs three complementary indices: Partition Coefficient (PC) quantifying membership overlap; Classification Entropy (CE) measuring partition fuzziness; and Xie-Beni Index (XB) evaluating cluster compactness and separation. F1 scores assess classification performance across identified clusters.

### 3.5. Cluster-HRI integration and exploratory spatial data analysis

Fuzzy clustering outcomes integrate with HRI through spatial aggregation at H3 hexagonal level. Both clustering assignments and HRI values are computed independently at establishment level, then merged using H3 indexing. For each cell, the most prevalent cluster becomes dominant; HRI values are aggregated using mean and standard deviation. One-way ANOVA and Kruskal-Wallis tests assess whether HRI values differ significantly across dominant cluster types. Effect sizes (η ^2^) quantify the proportion of HRI variance associated with cluster membership. Significant associations indicate systematic co-variation but do not establish that cluster characteristics cause risk differences.

Because the HRI vulnerability component incorporates economic and operational characteristics that partially overlap with cluster-defining variables, a component decomposition procedure is applied to distinguish genuine spatial risk variation from index construction artifacts. The procedure operates in three steps. First, separate one-way ANOVAs test each HRI component (hazard, exposure, vulnerability) individually against cluster membership, identifying which dimensions drive the observed composite HRI differences. Second, a reduced-form HRI is computed by excluding the vulnerability component entirely, and the cluster-HRI association is re-tested on this hazard-exposure-only index. If an association survives vulnerability removal, it reflects spatial positioning rather than scoring circularity. Third, extreme-case sensitivity tests the association under pure multiplicative (α = 1) and pure additive (α = 0) formulations, verifying that findings are not contingent on the specific balance between interaction and independent effects in the dual-component formula.

To characterize the spatial organization of HRI distributions, an Exploratory Spatial Data Analysis (ESDA) framework ( Anselin 1999) is employed comprising one global and two local statistics. Global Moran’s I is computed for each clustering scenario and sector. Moran’s I measures spatial autocorrelation (the degree to which nearby H3 cells exhibit similar HRI values) on a scale from −1 (perfect dispersion) through 0 (random) to +1 (perfect clustering). Statistical significance is assessed via permutation-based inference, testing whether observed spatial patterns exceed what random placement would produce.

The global statistic is complemented with two local indicators that decompose spatial autocorrelation to individual cells. Local Indicators of Spatial Association (LISA; Anselin 1995) partition the global Moran’s I into per-cell contributions (Ii), classifying each H3 cell as spatially clustered (Ii > 0, indicating similar HRI values among neighbors) or spatially outlying (Ii < 0, indicating dissimilar HRI values). Significance is assessed via permutation inference at p < 0.05. Getis-Ord Gi* ( Getis and Ord 1992) identifies statistically significant hotspots (high-HRI clusters) and cold spots (low-HRI clusters) using standardized Z-scores. Both local statistics use the same distance-based spatial weights matrix (25 km threshold, binary style) as the global test, ensuring consistent neighborhood definitions across the ESDA framework. The 25 km threshold reflects the spatial extent of major tourism destination clusters along this coastline (e.g., the Rimini-Cattolica corridor spans approximately 40 km, the Venice-Jesolo corridor approximately 30 km), ensuring that cells within the same destination system are treated as neighbors while avoiding connections between geographically distinct tourism zones.

## 4. Results

### 4.1. Descriptive overview

The analysis encompasses 10,022 hospitality establishments distributed across coastal Veneto and Emilia-Romagna ( Table 1). Mean elevation values for accommodations (M = 1.47 m, SD = 1.42) and services (M = 1.83 m, SD = 2.19) confirm low-lying topography, with negative minimum elevations indicating subsidence-affected areas. Coastal concentration is pronounced: 77.3% of establishments are located within 1 km of the sea, 79.4% within 2 km, with a median coastline distance of 337 meters. This represents the first comprehensive spatial characterization of hospitality distribution for this region.

**Table 1.** Descriptive statistics and frequency distributions for hospitality establishments by sector.

Panel A: Continuous variables									
	Accommodations (n = 5,030)				Services (n = 4,992)			
Variable	Mean	SD	Min	Max	Mean	SD	Min	Max
Elevation (m)	1.47	1.42	−2.42	11	1.83	2.19	−3.47	11
Distance to sea (m)	1109	1782	6	16041	977	1431	10	14768
Distance to waterway (m)	1031	1294	0.02	15048	1084	1565	0.06	15031
Distance to estuary (m)	11127	21609	6	78435	977	1431	10	14768
SLR projection RCP4.5 2030 (m)	1.36	1.40	−2.65	10.90	1.71	2.16	−6.04	10.90
Building height (m)	9.86	4.35	−1	30.51	8.26	4.4	−1.00	31.68
Price (€)	204	276	1	4536	14	11	0.25	270

Panel B: Establishment types				
	Accommodations (n = 5,030)		Services (n = 4,992)	
Type	n	%	n	%
Hotels	3480	69.20	—	—
Holiday rentals	1286	25.60	—	—
B&B	93	1.80	—	—
Agritourism	76	1.50	—	—
Other accommodation	95	1.90	—	—
Restaurants	—	—	3131	62.70
Bars/cafés	—	—	1244	24.90
Shops/retail	—	—	207	4.10
Other services	—	—	410	8.20

Panel C: Beach access									
	Accommodations (n = 5,030)				Services (n = 4,992)			
Category			n	%			n	%
General access			2201	43.80			1616	32.40
Private access			1453	28.90			—	—
No direct access			1016	20.20			3059	61.30
N/A			360	7.20			317	6.30

The two sectors differ in spatial distribution. Accommodations show substantial variability in estuarine proximity (M = 11,126.73 m, SD = 21,608.90), spanning immediate coastal zones to inland areas including the Venice lagoon system. Services cluster closer to water bodies (M = 977.10 m, SD = 1,431.33). Hotels comprise 69.2% of accommodations (n = 3,480), followed by holiday rentals at 25.6% (n = 1,286). Restaurants account for 62.7% of services (n = 3,131). Beach access shows trimodal distribution for accommodations: 43.8% general access, 28.9% private access, 20.2% no direct access ( Table 1).

### 4.2. HRI distributions, sensitivity, and external validation

**Figure 2.**
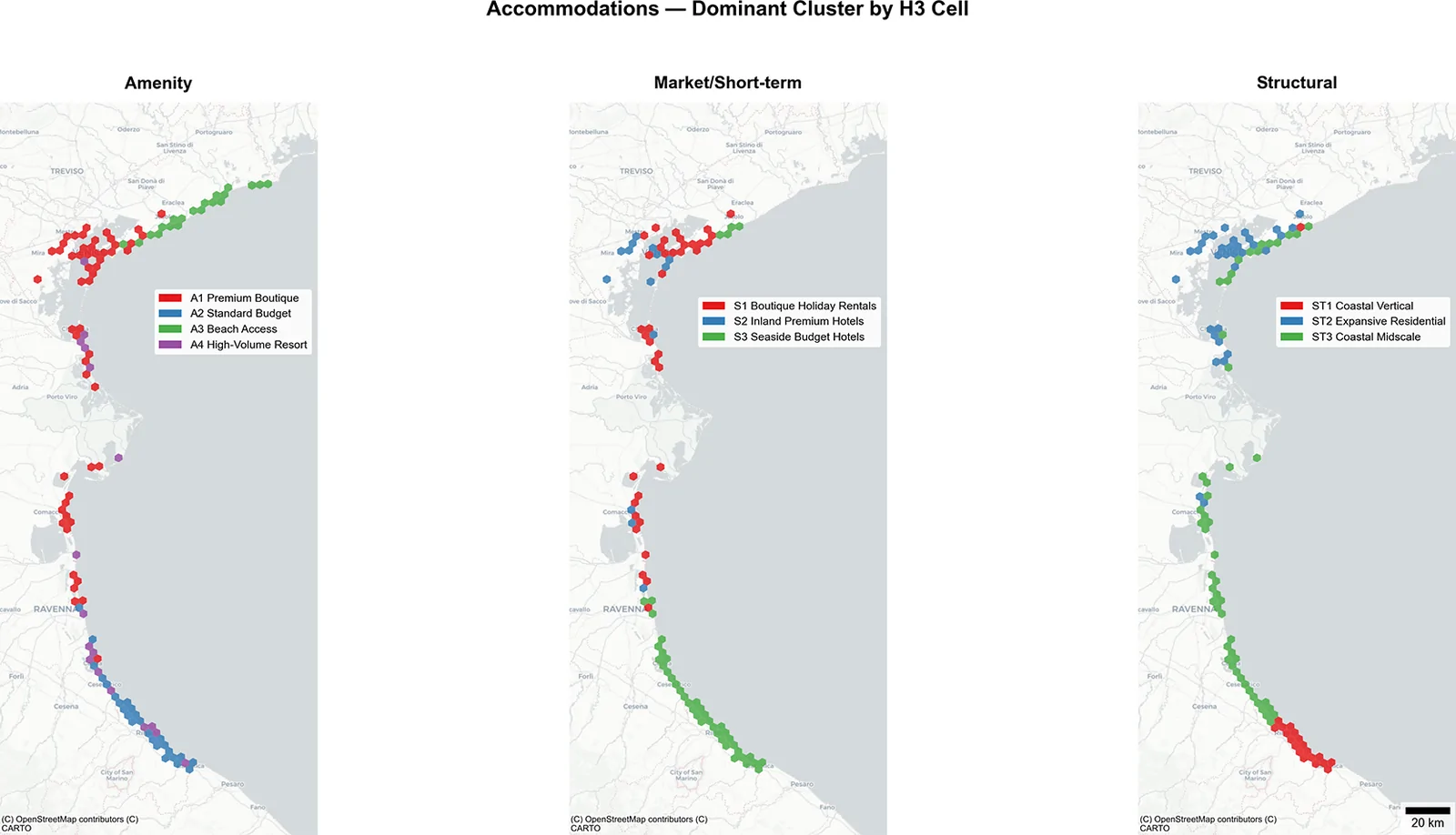
Accommodation HRI component maps under the Structural clustering scenario (104 H3 cells): Hazard, Exposure, Vulnerability, and Standardized HRI.

The accommodation HRI ( Figure 2) exhibits a pronounced north-south gradient in hazard scores, with elevated values (75–100%) concentrated in northern Veneto corresponding to low-elevation Venice lagoon areas. Exposure distributes more uniformly (50–75%) along the coastal corridor. Vulnerability scores (25–75%) reflect adaptive capacity differentiation across establishment types. The standardized HRI concentrates high-risk scores (75–100%) in northern coastal sections, moderate-to-high levels (50–75%) centrally, with variable patterns southward.

**Figure 3.**
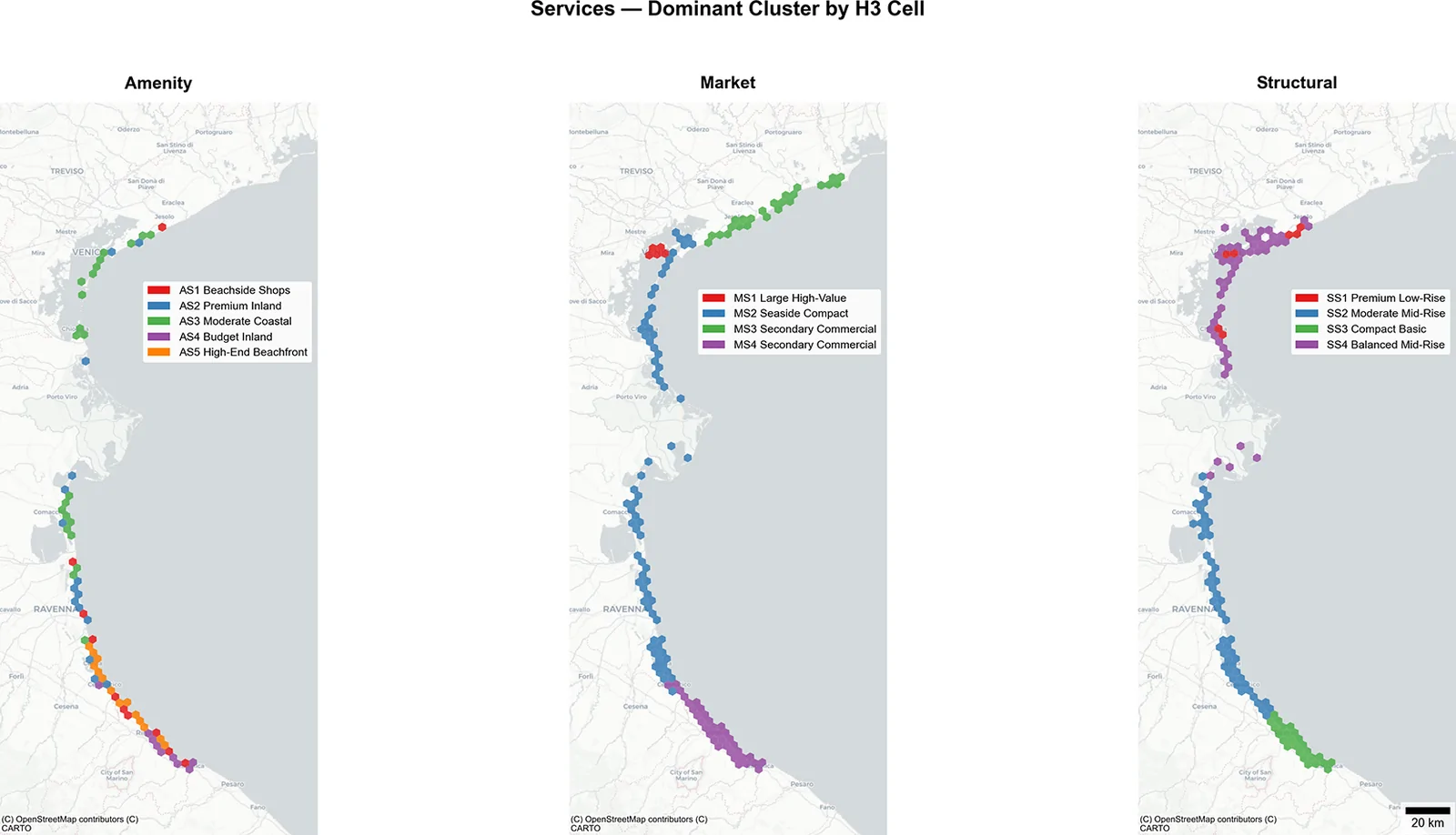
Service HRI component maps under the Structural clustering scenario (130 H3 cells): Hazard, Exposure, Vulnerability, and Standardized HRI.

The service HRI ( Figure 3) shows marked spatial heterogeneity, with elevated hazard clusters (75–100%) in central coastal segments. Exposure intensifies in urban centers. High-risk HRI clusters (75–100%) distribute along the coastline with consistent moderate-to-high levels (50–75%) centrally. Figures Figure 2 Figure 3 map HRI components under the Structural clustering scenario, which produces statistically significant spatial autocorrelation for both sectors (Moran’s I = 0.115, p < 0.001 for accommodations; 0.293, p < 0.001 for services) and yields the strongest cluster-HRI association (η ^2^ = 0.152 and 0.221, respectively). This scenario covers 104 accommodation and 130 service H3 cells, representing 92% of establishments in each sector. Hazard, exposure, and vulnerability scores are computed independently of cluster membership; the clustering scenario determines only which subset of H3 cells is represented.

Sensitivity analysis across 25 constrained parameter combinations (α + β = 1, aH + aE + aV = 1) confirms methodological stability (Extended Data Figure 1). Accommodation mean HRI ranges from 0.306 to 0.443 (CV = 10.1%); services range from 0.288 to 0.401 (CV = 9.4%). The broader parameter space produces wider ranges than a narrower grid would, but the coefficient of variation remains below conventional instability thresholds. The index structure holds across the full space of proportionally valid weight combinations. This CV metric captures stability of mean index levels; rank-order stability across parameterizations was not formally tested, though the component decomposition results (Section 4.4) are invariant to weight selection because they test components individually.

Beyond internal stability, external validation tests whether the HRI identifies establishments that actually experienced flood conditions. Within the three Copernicus EMS Areas of Interest (Venice, Comacchio, Ravenna), the analysis identifies 2,801 establishments: 1,367 accommodations and 1,434 services. Applying the 50 m flood-proximity buffer classifies 504 accommodations (36.9%) and 317 services (22.1%) as flood-proximate, yielding a combined validation sample of 821 flood-proximate and 1,980 non-proximate establishments ( Table 2).

**Table 2.** Establishment-level HRI by flood proximity (50 m buffer, AOI-restricted): sample size, median and mean HRI, Mann-Whitney W statistic, p-value, and rank-biserial correlation by sector.

Sector	Group	n	Median HRI	Mean HRI	SD	W	p	r
Accommodations	Flood-proximate	504	0.361	0.368	0.108	268,933	<0.001	0.24
Accommodations	Not proximate	863	0.315	0.323	0.105			
Services	Flood-proximate	317	0.411	0.445	0.146	198,333	<0.001	0.12
Services	Not proximate	1,117	0.405	0.408	0.139			
Pooled	Flood-proximate	821	0.376	0.398	0.131	903,503	<0.001	0.11
Pooled	Not proximate	1,980	0.359	0.373	0.130			

Flood-proximate establishments exhibit significantly higher HRI than non-proximate establishments in both sectors (accommodations: p < 0.001, r = 0.24; services: p < 0.001, r = 0.12) and in the pooled sample (p < 0.001, r = 0.11). Effect sizes are small to medium, consistent with expectations: the HRI integrates multiple dimensions beyond flood exposure alone, and the comparison is restricted to establishments within the same monitored coastal zones rather than contrasting coastal against inland areas (Extended Data Figure 2).

Component decomposition reveals that the HRI difference is driven by the exposure dimension (Extended Data Figure 3). Physical exposure scores are +0.083 (accommodations) and + 0.088 (services) higher for flood-proximate establishments, reflecting lower elevation and closer coastal proximity. Composite exposure, which adds spatial density, shows an even larger gap (+0.126 and + 0.139 respectively). Hazard scores are essentially identical between groups (difference < 0.01 for accommodations; −0.04 for services), as expected within the same coastal segments where sea-level rise projections are spatially uniform. Vulnerability scores are slightly lower for flood-proximate establishments (−0.007 accommodations, −0.016 services). Flood-proximate establishments within the AOIs are on average more expensive (accommodations: median €212 vs €138; services: median €20 vs €12) and taller (accommodations: 11.2 m vs 9.2 m; services: 11.7 m vs 7.5 m), both of which reduce vulnerability scores through the adaptive capacity and building height components (Extended Data Figure 4). This raises the question of whether the adaptive capacity proxy simply echoes spatial positioning, with higher prices in flood-prone locations reflecting location desirability rather than genuine economic resources for coping with hazards. Location variables (coastal distance, elevation) explain less than 8% of price variance (R ^2^ = 0.019 for accommodations, 0.074 for services), indicating that the price premium of flood-proximate establishments primarily reflects non-spatial economic characteristics. The adaptive capacity proxy thus captures genuine economic differentiation largely independent of spatial positioning, though whether these economic resources translate into actual flood preparedness cannot be determined from cross-sectional data ( Mortreux and Barnett 2017). The component decomposition approach addresses the residual confounding by testing each HRI dimension independently against cluster membership, separating exposure-driven from vulnerability-driven risk variation.

**Figure 4.**
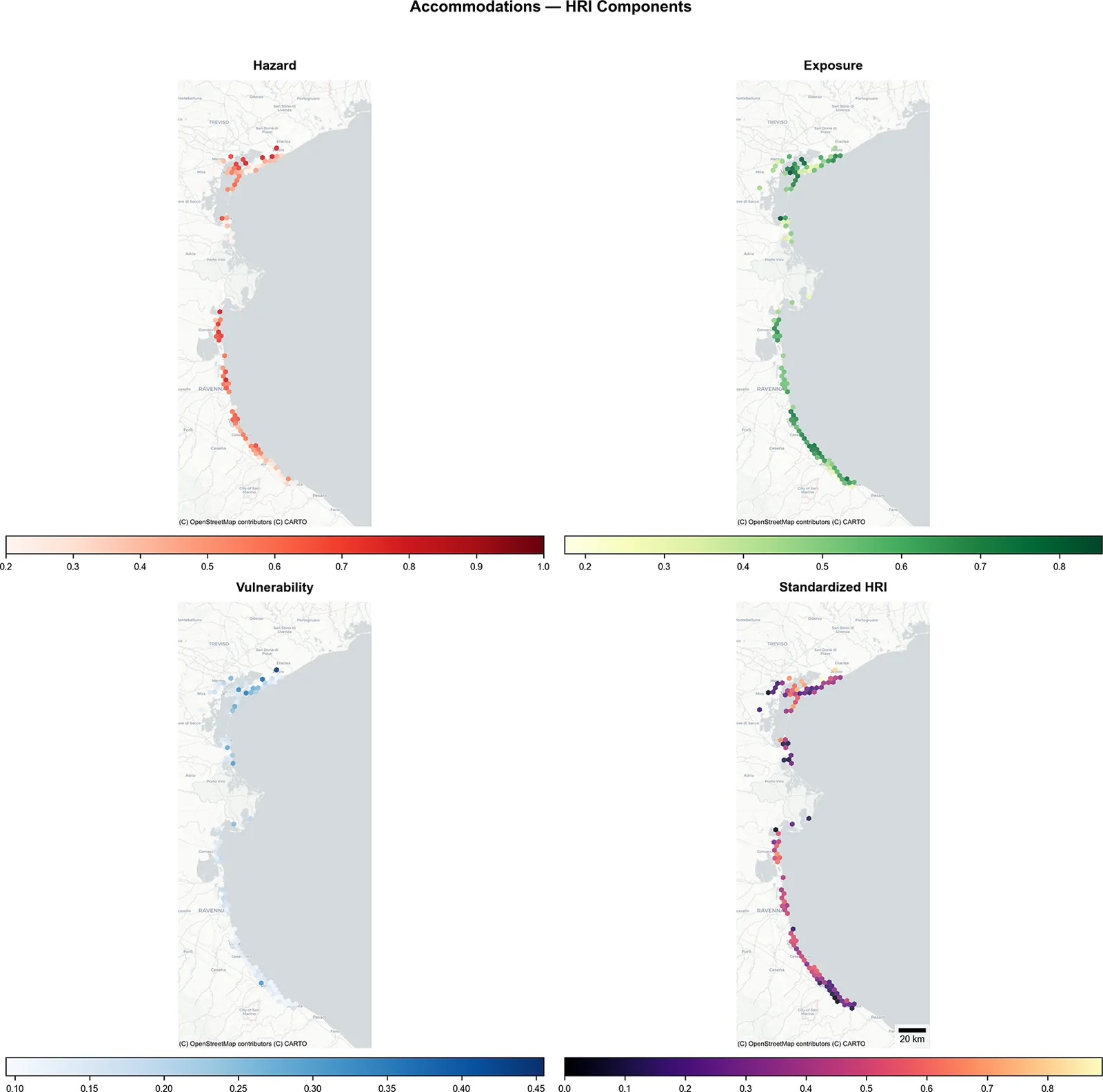
Accommodation clustering spatial patterns showing H3 dominant cluster assignments for Amenity (4 clusters), Market/Short-Term (3 clusters), and Structural (3 clusters) scenarios.

### 4.3. Cluster analysis

Clustering analyses use subsets of the full database due to variable availability requirements: sample sizes vary by scenario (accommodation scenarios: n = 1,903 to 3,133; service scenarios: n = 461 to 4,837). The service amenity scenario uses only 9% of the service sample (n = 461), limiting generalizability for that specific analysis. Synergy analyses include only H3 cells with dominant cluster assignments; coverage ranges from 43% (service amenity, 70 of 161 cells) to 82% (service market, 132 of 161 cells) for services and from 64% (accommodation market, 93 of 145 cells) to 81% (accommodation amenity, 117 of 145 cells) for accommodations. Cells lacking dominant assignments are excluded because no establishments in those cells met the clustering subset’s variable availability requirements.

Fuzzy clustering validation demonstrates acceptable quality across scenarios (Extended Data Table 1). Accommodation amenity clustering yields PC = 0.592 and XB = 0.832, indicating moderate cluster separation, with F1 scores ranging from 0.566 to 0.868 across four clusters. Service amenity clustering shows PC = 0.516 and XB = 0.662, with F1 scores from 0.533 to 0.819 across five clusters. Services achieve tighter cluster compactness (lower XB) while accommodations show higher average classification performance. These metrics support the fuzzy clustering framework’s reliability for typology identification.

4.3.1. Accommodation typologies

The three clustering scenarios identify distinct accommodation typologies. Amenity clustering (n = 2,260) identifies four typologies with north-south spatial differentiation ( Figure 4, Amenity panel): A1 (n = 851) premium boutique establishments in heritage locations (€291/night, 14.8 rooms mean); A2 (n = 625) standard budget accommodations along the Emilia-Romagna coast (€194/night, 23.8 rooms); A3 (n = 416) exclusive beach-access establishments along the Jesolo-Caorle corridor (€202/night, 41.6 rooms); and A4 (n = 368) high-volume resort properties in family-tourism destinations (€188/night, 63 rooms). This scenario captures the adaptive capacity dimension: the price and rating differentiation across clusters reflects the economic resources available for risk mitigation.

Market clustering (n = 1,903) identifies three typologies distinguished by coastal positioning, operationalizing the bid-rent sorting mechanism: S1 (n = 414) boutique holiday rentals in the Venice-Lido area (€275/night, 2,226 m from coast); S2 (n = 399) inland premium hotels in Venice terraferma and Ravenna hinterland (€334/night, 3,925 m); and S3 (n = 1,090) seaside budget hotels dominating the Rimini-Cattolica corridor (€179/night, 241 m).

Structural clustering (n = 3,133) identifies three morphological patterns that distinguish the infrastructure types central to the platform restructuring discussion: ST1 (n = 1,259) vertical high-capacity establishments along the urbanized Emilia-Romagna coast (12.1 m height, 273 m from coast, 32 rooms); ST2 (n = 840) expansive residential properties in the Venice lagoon, Po Delta, and mainland (3,576 m from coast, 447 m ^2^ footprint, median 3 rooms), representing converted residential properties including agritourism and short-term rentals; and ST3 (n = 1,034) coastal midscale establishments in transitional zones (285 m from coast).

4.3.2. Service typologies

Service clustering scenarios reveal typologies shaped by the agglomeration dynamics and coastal positioning. Amenity clustering (n = 461, 9% of service sample) identifies five typologies with coastal-inland differentiation: AS1 (n = 74) beachside shops along Venice Lido; AS2 (n = 112) premium inland restaurants in historic centers (€13.04 mean, 600 m from coast); AS3 (n = 86) moderate coastal restaurants (€10.82, 299 m); AS4 (n = 79) budget inland restaurants (€8.49, 700 m); and AS5 (n = 110) high-end beachfront restaurants (€11.73, 180 m). Given the small sample, these results should be interpreted cautiously.

Market clustering (n = 4,837) identifies four typologies differentiated primarily by capitalization intensity. MS1 comprises large high-value establishments in prime commercial districts. MS2 (n = 1,868) groups seaside compact establishments concentrated along beachfront strips, characterized by smaller footprints (292 m ^2^ mean) but premium positioning. MS3 and MS4 encompass establishments distributed across secondary commercial positions.

**Figure 5.**
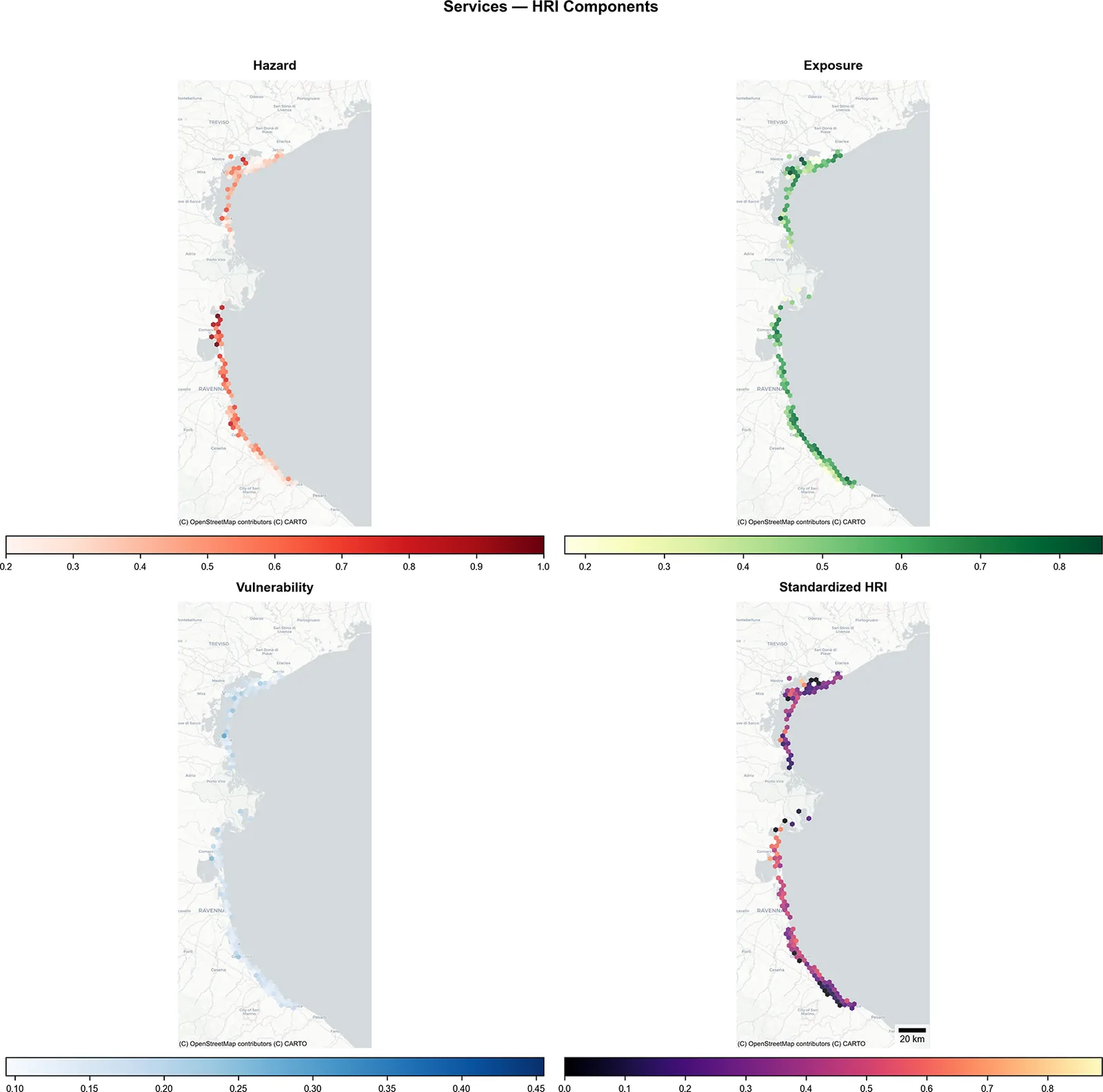
Service clustering spatial patterns showing H3 dominant cluster assignments for Amenity (5 clusters), Market (4 clusters), and Structural (4 clusters) scenarios.

Structural clustering (n = 4,030) identifies four development patterns. SS1 (n = 756) comprises premium low-rise establishments in heritage-protected zones with constrained vertical development (5.2 m mean height). SS2 (n = 1,247), the largest segment, groups moderate mid-rise establishments with intermediate characteristics (6.8 m mean height, 322 m ^2^ mean footprint). SS3 encompasses compact basic establishments in secondary locations. SS4 (n = 1,344) groups balanced mid-rise establishments predominating along the urbanized Emilia-Romagna coast ( Figure 5).

### 4.4. Cluster-HRI associations and component decomposition

Statistical analysis examines whether HRI values differ systematically across dominant cluster types within H3 hexagonal cells ( Table 3). For accommodations, structural clustering shows the strongest association with HRI (F = 9.084, p < 0.001, η ^2^ = 0.152). Cells dominated by ST2 (Expansive Residential) show mean HRI of 0.422 versus 0.231 for ST1 (Coastal Vertical High-Capacity), a difference of 0.191 in standardized scores. Market clustering shows association at relaxed threshold (F = 2.574, p = 0.082, η ^2^ = 0.054), with S1 (Boutique Holiday Rentals) cells highest (0.441) and S2 (Inland Premium Hotels) lowest (0.339). Amenity clustering shows no significant association (F = 1.175, p = 0.322).

**Table 3.** Cluster-HRI association statistics: one-way ANOVA F-statistic, p-value, η ^2^ effect size, and mean HRI by dominant cluster for each clustering scenario and sector.

Panel A: One-way ANOVA results						
Sector	Scenario	k	df _1_, df _2_	F	p	η ^2^
Accommodations	Amenity	4	3, 113	1.175	0.322	0.03
Accommodations	Market	3	2, 90	2.574	0.082	0.054
Accommodations	Structural	3	2, 101	9.084	<0.001	0.152
Services	Amenity	5	4, 65	4.724	0.002	0.225
Services	Market	4	3, 128	3.159	0.027	0.069
Services	Structural	4	3, 126	11.95	<0.001	0.221

Panel B: Mean standardized HRI by dominant cluster (n = H3 cells)					
Sector	Scenario	Cluster	Mean HRI	SD	n
Accommodations	Amenity	A1	0.408	0.214	54
		A2	0.334	0.159	26
		A3	0.395	0.207	20
		A4	0.335	0.158	17
Accommodations	Market	S1	0.441	0.212	37
		S2	0.339	0.19	14
		S3	0.361	0.146	42
Accommodations	Structural	ST1	0.231	0.131	20
		ST2	0.422	0.235	33
		ST3	0.418	0.149	51
Services	Amenity	AS1	0.37	0.097	10
		AS2	0.405	0.171	15
		AS3	0.416	0.134	22
		AS4	0.222	0.106	9
		AS5	0.44	0.088	14
Services	Market	MS1	0.439	0.132	5
		MS2	0.385	0.182	69
		MS3	0.365	0.212	24
		MS4	0.277	0.155	34
Services	Structural	SS1	0.382	0.113	7
		SS2	0.44	0.144	50
		SS3	0.229	0.144	25
		SS4	0.297	0.185	48

Notes: k = number of clusters; η ^2^ = eta-squared effect size (SS_between/SS_total). Mean HRI values are standardized (0–1 scale). Kruskal-Wallis non-parametric tests confirm all ANOVA conclusions (see text). Full per-cluster statistics available in replication package.

For services, all clustering scenarios show significant associations at α = 0.05. Structural clustering yields the strongest association (F = 11.95, p < 0.001, η ^2^ = 0.222), with SS2 (Moderate Mid-Rise) cells scoring highest (HRI = 0.440). Amenity clustering demonstrates comparable effect size (F = 4.724, p = 0.002, η ^2^ = 0.225), with AS5 (High-End Beachfront) cells highest (0.440) and AS4 (Budget Inland) lowest (0.222). Market clustering shows a weaker but significant association (F = 3.159, p = 0.027, η ^2^ = 0.069), with MS1 (Large High-Value) cells highest (0.439, n = 5 cells; interpret with caution).

Kruskal-Wallis tests confirm all ANOVA conclusions: accommodation structural (χ ^2^ = 16.82, p < 0.001), service structural (χ ^2^ = 32.96, p < 0.001), service amenity (χ ^2^ = 14.56, p = 0.006), and service market (χ ^2^ = 9.15, p = 0.027) are significant; accommodation amenity (χ ^2^ = 3.05, p = 0.384) and market (χ ^2^ = 3.36, p = 0.187) are non-significant. Results are not artifacts of ANOVA distributional assumptions.

To assess whether these associations reflect genuine spatial risk differences or index construction circularity, each HRI component is tested against cluster membership independently ( Table 4).

**Table 4.** HRI component decomposition: separate ANOVA results testing hazard, exposure, vulnerability, and reduced-form HRI (without vulnerability) against cluster membership by scenario and sector.

Sector	Scenario	Component	F	p	η ^2^
Acc	Structural	Hazard	10.333	<0.001	0.170
Acc	Structural	Exposure	1.679	0.192	0.032
Acc	Structural	Vulnerability	4.527	0.013	0.082
Acc	Structural	HRI without vuln	7.012	0.001	0.122
Acc	Structural	Full HRI	9.084	<0.001	0.152
Acc	Market	Hazard	2.337	0.102	0.049
Acc	Market	Exposure	1.855	0.162	0.040
Acc	Market	Vulnerability	20.471	<0.001	0.313
Acc	Market	HRI without vuln	0.618	0.541	0.014
Acc	Market	Full HRI	2.574	0.082	0.054
Ser	Structural	Hazard	21.301	<0.001	0.337
Ser	Structural	Exposure	4.267	0.007	0.092
Ser	Structural	Vulnerability	4.486	0.005	0.097
Ser	Structural	HRI without vuln	13.267	<0.001	0.240
Ser	Structural	Full HRI	11.951	<0.001	0.222
Ser	Amenity	Hazard	3.949	0.006	0.196
Ser	Amenity	Exposure	7.317	<0.001	0.310
Ser	Amenity	Vulnerability	1.296	0.281	0.074
Ser	Amenity	HRI without vuln	4.654	0.002	0.223
Ser	Amenity	Full HRI	4.724	0.002	0.225

For accommodation structural clustering, the association survives vulnerability removal (η ^2^ = 0.122, p = 0.001), with hazard as the primary driver (η ^2^ = 0.170). For accommodation market clustering, vulnerability explains 31.3% of variance (η ^2^ = 0.313) while hazard-exposure without vulnerability explains only 1.4% (η ^2^ = 0.014, p = 0.541). For service clustering, both structural and amenity scenarios are robust to vulnerability removal, driven by hazard (η ^2^ = 0.337) and exposure (η ^2^ = 0.310) respectively. Extreme-case sensitivity (pure multiplicative α = 1 vs. pure additive α = 0) confirms accommodation structural patterns persist across all formulations (η ^2^ = 0.146–0.158, all p < 0.001), while market and amenity patterns remain non-significant regardless of formulation. These decomposition results depend on the input components (hazard geography, exposure gradients) rather than the specific aggregation formula; the dual-component formulation and simpler alternatives (e.g., geometric mean, weighted linear sum) would produce the same component-level patterns because the components themselves are invariant to the aggregation method.

### 4.5. Spatial Clustering of HRI Distributions

The ESDA framework (Section 3.5) quantifies spatial organization of HRI distributions through one global and two local statistics. Global Moran’s I statistics reveal contrasting spatial clustering between sectors. Accommodations demonstrate weak spatial autocorrelation (Moran’s I = 0.069–0.115, all p < 0.001), statistically significant but limited at regional scales. Services exhibit two- to four-fold stronger spatial organization (Moran’s I = 0.133–0.293, all p < 10 ^−12^).

This divergence indicates that services concentrate in spatial clusters (commercial districts, tourism corridors) while accommodations distribute more heterogeneously across the study area. Both patterns achieve statistical significance across all scenarios, confirming genuine spatial organization rather than random distribution.

**Figure 6.**
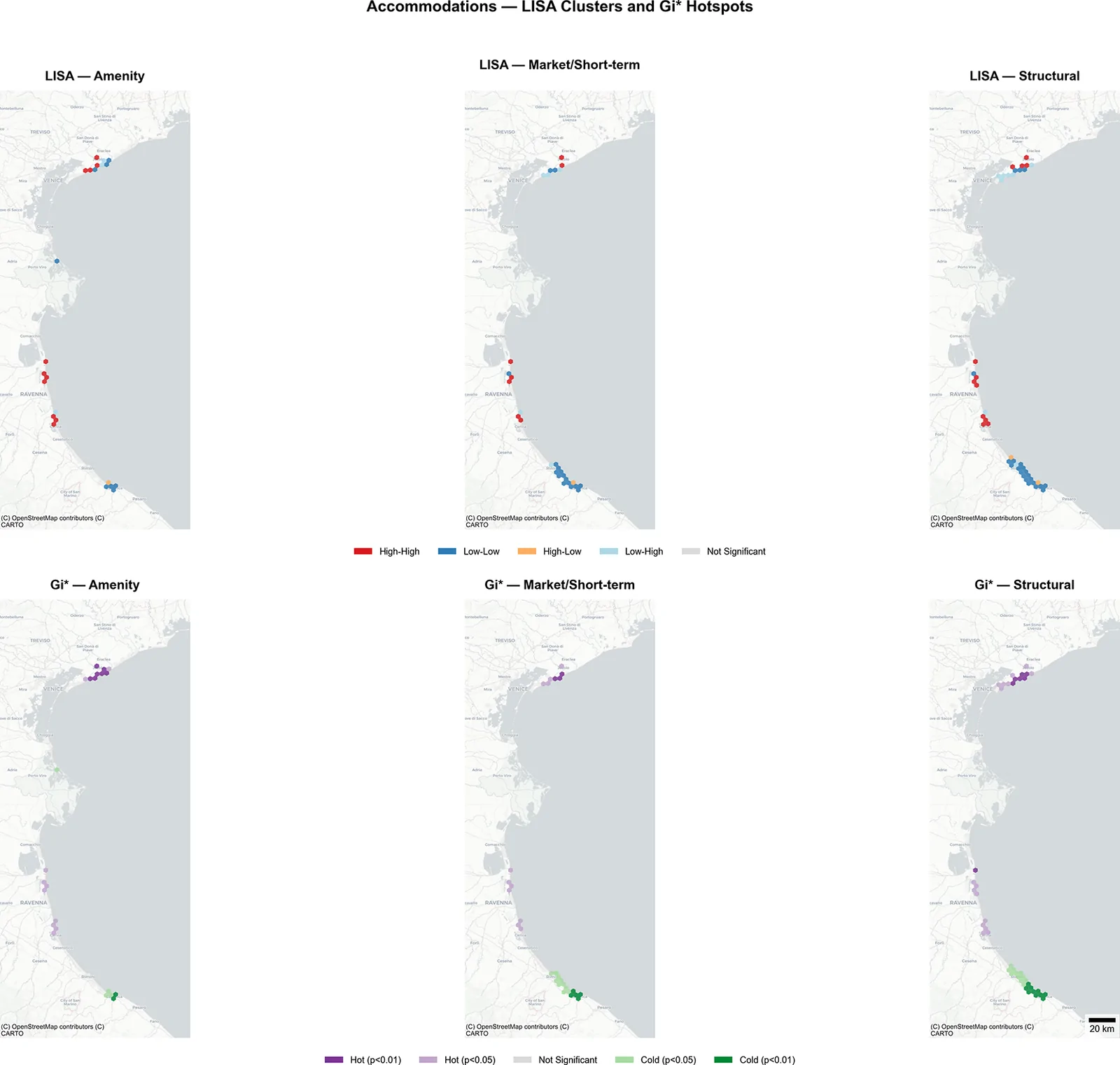
Local spatial statistics for accommodation HRI: LISA cluster maps (above) and Getis-Ord Gi* hotspot/coldspot maps (below) across Amenity, Market/Short-Term, and Structural scenarios. LISA: High-High (red), Low-Low (blue), High-Low (orange), Low-High (light blue). Gi*: Hot Spot p < 0.01 (purple), Hot Spot p < 0.05 (light purple), Cold Spot p < 0.05 (light green), Cold Spot p < 0.01 (dark green).

Local spatial statistics confirm and localize this sectoral divergence. LISA identifies 47.7–57.1% of service cells as exhibiting statistically significant local spatial autocorrelation (p < 0.05), compared with 19.7–42.3% for accommodations (Extended Data Table 2). Across both sectors, spatially clustered cells (positive Ii, indicating similar HRI values among neighbors) substantially outnumber spatial outliers (negative Ii, indicating dissimilar values), indicating that HRI clustering dominates over isolated high- or low-risk cells. Accommodation structural clustering shows the highest proportion of significant cells among accommodation scenarios (42.3%, with 34 clusters and 10 outliers) as shown in Figure 6.

**Figure 7.**
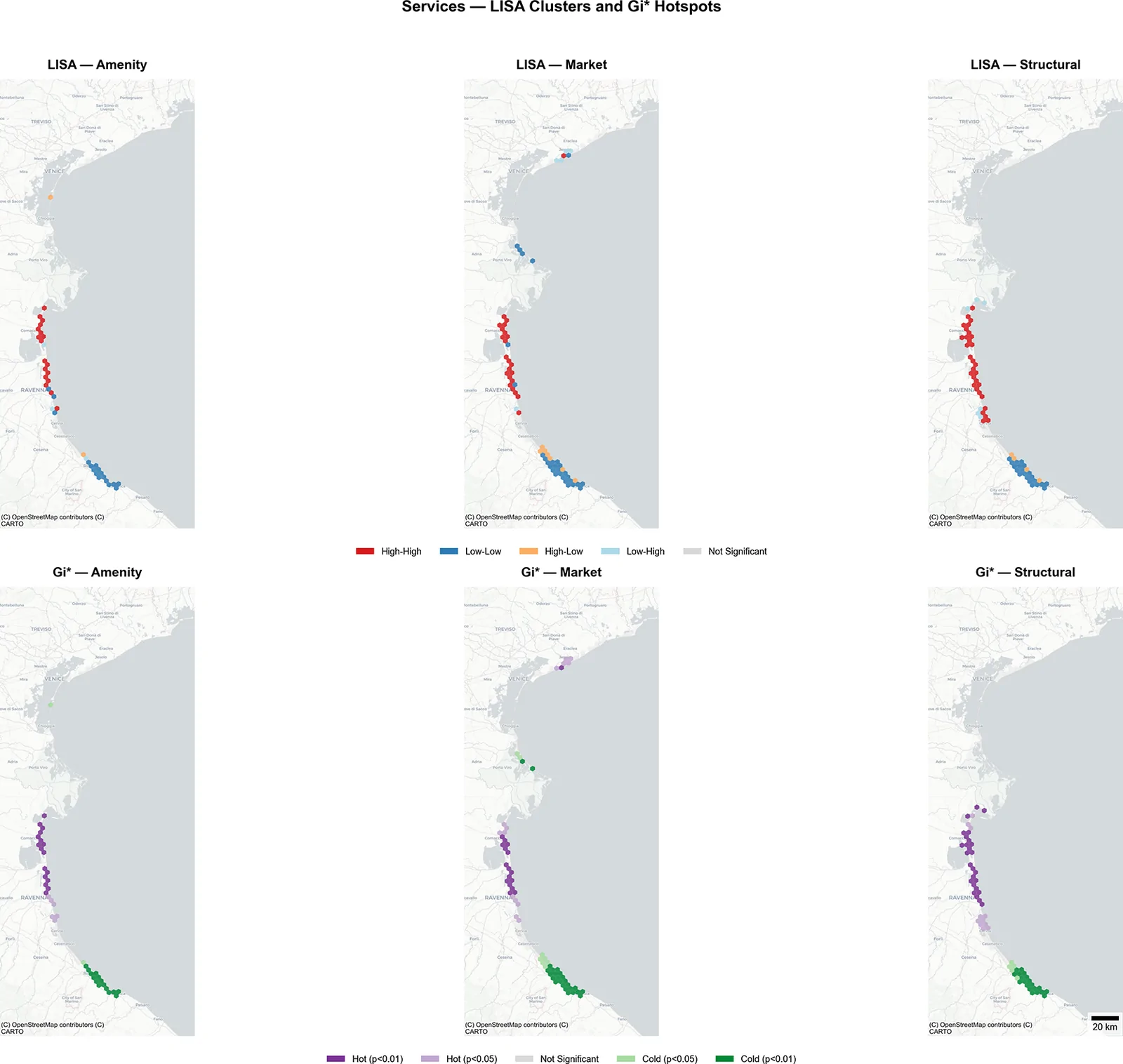
Local spatial statistics for service HRI: LISA cluster maps (above) and Getis-Ord Gi* hotspot/coldspot maps (below) across Amenity, Market, and Structural scenarios. Same classification scheme as Figure 6.

Getis-Ord Gi* analysis reveals scenario-specific hotspot asymmetries. Accommodation amenity clustering shows a pronounced asymmetry: 17 hotspots versus only 6 cold spots, indicating that high-HRI clustering is more spatially concentrated than low-HRI clustering in this scenario. Accommodation market and structural scenarios show more balanced hotspot-cold spot distributions (14:15 and 23:21 respectively). For services, structural clustering produces the strongest individual hotspot (Gi* = 5.01) and the largest hotspot count (37), confirming that service risk concentrates in geographically specific clusters. Services consistently show more hotspots than accommodations across all scenarios, reinforcing the global Moran’s I finding that service HRI exhibits stronger spatial organization ( Figure 7).

## 5. Discussion

### 5.1. Sectoral divergence and component decomposition

The analysis reveals that accommodation and service establishments exhibit fundamentally different patterns of spatial risk variation, a divergence sharper than a simple amenity-hazard gradient would predict. Cluster membership accounts for 5–22% of HRI variation (η ^2^ range: 0.054–0.225), with the remaining variance reflecting spatial position, local hazard conditions, and establishment-specific characteristics outside the clustering dimensions. The modest effect sizes are expected given that clusters capture typological groupings while HRI integrates fine-grained spatial variables, but the pattern of which associations survive component decomposition proves more informative than the magnitude alone.

For accommodations, the most pronounced HRI differentiation occurs across establishment types, not coastal distance gradients. Structural clustering shows the strongest association (η ^2^ = 0.152). Converted residential properties (ST2, HRI = 0.422) score substantially higher than vertical high-capacity coastal hotels (ST1, HRI = 0.231). This finding complicates the expectation that beach access should elevate risk regardless of market positioning. For services, that expectation holds: high-end beachfront establishments (AS5, HRI = 0.440) score highest despite premium pricing, while budget inland services (AS4, HRI = 0.222) score lowest. For accommodations, however, beachfront hotels score lower than inland converted properties because the Venice lagoon system creates multi-directional hazard pathways that make Euclidean coastal distance a poor proxy for flood exposure. The amenity-hazard coupling operates in both sectors, but through different spatial mechanisms: for services, through direct coastal proximity; for accommodations, through the lagoonal geography that concentrates converted residential stock.

The component decomposition discriminates among candidate interpretations and provides the diagnostic evidence needed to disentangle the overlapping effects of hazard geography, exposure, and adaptive capacity. For structural clustering, the association survives vulnerability removal: hazard alone is the primary driver, with ST2 cells averaging substantially higher hazard scores than ST1 cells. This supports a lagoon hazard geography interpretation: structural clusters map onto distinct hazard geographies rather than simply reflecting vulnerability score differences. The pattern is consistent with platform-driven residential conversion concentrating tourism activity in hazard-proximate locations, though the cross-sectional design cannot distinguish whether conversion causes higher vulnerability or whether high-risk locations are simply more likely to contain converted properties. For market clustering, the association is driven by the vulnerability component: removing vulnerability renders the cluster-HRI relationship non-significant (η ^2^ drops from 0.054 to 0.014). This indicates that market-based clusters differentiate primarily by economic characteristics that the vulnerability component captures, rather than by distinct hazard geographies, a finding that validates the component decomposition as a diagnostic tool for distinguishing index construction effects from genuine spatial risk variation. Two additional interpretations (infrastructure inadequacy in converted residential properties, cf. Braswell et al. 2022; and selection effects driving higher-risk properties into short-term rental markets) remain plausible but cannot be assessed from these data alone. The external validation provides complementary evidence: establishments within 50 m of observed Copernicus EMS flood extents score significantly higher on the HRI, and this difference is driven by the exposure component (+0.08 to +0.14) while vulnerability remains independent of flood status. This pattern confirms that the exposure component captures genuine flood-relevant spatial positioning rather than index construction artifacts.

For services, HRI scores correlate consistently with coastal proximity across structural and market scenarios, which together cover 80–82% of H3 cells, though the amenity results derive from only 70 cells (43% coverage, n = 461 establishments) and should be interpreted as indicative rather than definitive. Structural and market patterns, based on substantially larger samples, reflect genuine spatial positioning (hazard geography and exposure respectively) rather than vulnerability circularity. This is where the adaptive capacity expectation meets its clearest test: if economic resources reduce net vulnerability, premium beachfront services should score lower than budget beachfront services. Instead, location effects dominate adaptive capacity effects. Premium pricing does not offset exposure-driven risk, a finding consistent with amenity-hazard coupling where the same coastal positioning that generates revenue simultaneously configures hazard exposure. For inland establishments, the expected adaptive capacity gradient does appear: budget inland services (AS4) show the lowest HRI scores. The economic dimension of vulnerability thus operates where exposure pressure is moderate, but is overwhelmed where exposure is high.

The two sectors also diverge in their spatial organization, as the location logic framework predicted. Services show two- to four-fold stronger spatial autocorrelation (Moran’s I: 0.133–0.293 vs. 0.069–0.115), consistent with agglomeration-driven clustering producing tighter spatial concentration than the amenity-driven sorting that characterizes accommodation. Local spatial statistics (LISA, Gi*) confirm this is not merely a global average effect: 47.7–57.1% of service cells show significant local clustering versus 19.7–42.3% for accommodations, with service structural clustering producing the strongest individual hotspot (Gi* = 5.01). Accommodation market and amenity clustering do not predict risk independently of hazard geography, while structural and all service patterns survive vulnerability removal. This asymmetry reinforces the interpretation of services as spatially organized by agglomeration and accommodations as differentiated by establishment type.

By measuring building characteristics (height, footprint, structural typology) as indicators of development-embedded vulnerability, the framework moves partially beyond treating the built environment as a static exposure surface. The component decomposition demonstrates where this approach succeeds: structural clustering captures genuine hazard geographies that reflect decades of development decisions. The decomposition also successfully identifies which associations are artifacts: the market-based cluster-HRI association reflects scoring overlap between adaptive capacity proxies and establishment type rather than independent spatial risk variation, a diagnostic result that demonstrates the value of component-level testing for composite index studies more broadly.

### 5.2. Development trajectories and north-south differentiation

The spatial analysis identifies pronounced north-south differentiation that reflects distinct tourism development trajectories. The integration of risk assessment with spatial land-use analysis responds to a long-recognized need in disaster risk management ( Menoni and Pergalani 1996), here applied to the specific context of coastal hospitality.

Northern Veneto, spanning from the Venice lagoon through the Po Delta (approximately 44.8°N to 45.5°N), concentrates converted residential accommodations. The ST2 cluster appears prominently in Venice mainland, Po Delta interior, and lagoon-adjacent areas. These properties face compound hazard exposure: Adriatic storm surges, river discharge, astronomical tides, and progressive land subsidence interact simultaneously ( Zanchettin et al. 2021; Tosi et al. 2013).

Southern Emilia-Romagna’s Rimini-Cattolica corridor (approximately 43.9°N to 44.3°N) concentrates beachfront service establishments. The 40-kilometer coastal strip hosts the highest density of AS5 (High-End Beachfront) services, reflecting decades of coordinated mass-tourism development oriented around beach access ( Perini et al. 2017; Armaroli et al. 2012).

This geographic bifurcation reflects divergent historical development trajectories. Northern Veneto’s tourism economy evolved around Venice’s existing urban fabric, expanding through residential conversion as platform-mediated accommodation enabled private homeowners to enter hospitality markets ( Gutiérrez et al. 2017; Cocola-Gant 2016). Southern Emilia-Romagna developed through coordinated post-war investment producing standardized hotel infrastructure and organized beach concession systems along a coastline where 93 km (71%) is extensively urbanized ( Armaroli et al. 2019; Perini et al. 2017). Both trajectories illustrate what Braswell et al. (2022) term “creeping disaster,” with historical coastal development progressively concentrating assets in hazard-prone locations and each building cycle extending the exposure frontier. In the south, coastal protection infrastructure may have further reinforced this pattern through the safe development paradox: flood defences encourage additional development in protected zones, increasing net risk even as per-unit protection improves ( Brody et al. 2011).

The accommodation sector finding connects directly to platform accommodation geography. The concentration of converted residential properties (ST2) in the Venice lagoon system mirrors documented spatial patterns of Airbnb proliferation in historic and waterfront districts ( Gutiérrez et al. 2017; Cocola-Gant 2016; Wachsmuth and Weisler 2018). Amenity-driven spatial sorting, with platform accommodation gravitating toward locations with high experiential value, co-occurs in the data with elevated hazard scores. Buildings situated near water, in historic settings, and in neighborhoods valued for ambiance are also buildings exposed to lagoonal flooding, subsidence, and multi-directional storm surge.

This pattern of hazard-amenity co-occurrence is not unique to the Northern Adriatic but likely characterizes many coastal and waterfront tourism destinations where the features that attract visitors (proximity to water, historic architecture, scenic settings) are the same features that produce hazard exposure. At the Mediterranean scale, where urban extent in low-lying coastal areas is projected to increase by 160% ( Wolff et al. 2020), development patterns may ultimately determine coastal flood exposure more than sea-level rise itself ( Wang et al. 2025). The historical embedding of these spatial configurations within decades of accumulated development decisions suggests they are resistant to rapid change. Where buildings have been converted to tourism use, where infrastructure has been oriented around beach access, and where economic livelihoods depend on waterfront positioning, the spatial patterns documented here are unlikely to shift through short-term interventions. Understanding how development trajectories produce these durable vulnerability configurations is a prerequisite for designing adaptation strategies that account for path dependence rather than assuming spatial flexibility.

### 5.3. Limitations

Four limitations constrain interpretation. First, the cross-sectional design documents associations but cannot establish whether infrastructure type influences vulnerability or whether higher-risk locations simply attract certain establishment types ( Atzori and Fyall 2018). Second, Euclidean distance to the Adriatic Sea poorly captures flood exposure in the Venice lagoon system, where multi-directional hazard pathways mean that properties classified as “inland” may face substantial flood risk. Third, the accommodation typology assumes a sharp distinction between converted residential properties and purpose-built hotels, but professionalization of the platform sector may attenuate this boundary, as professional Airbnb hosts concentrate in central touristic areas following hotel-like spatial patterns ( Gyódi 2023). Fourth, the adaptive capacity proxy captures primarily the economic dimension, while adaptive capacity also resides in social capital, networks, and institutional trust ( Adger 2003), and the relationship between capacity and action is not deterministic ( Mortreux and Barnett 2017).

### 5.4. Future research directions

The observed patterns generate four testable hypotheses: (1) converted residential properties exhibit higher actual flood vulnerability due to infrastructure differences, testable through validation against flood damage or insurance data; (2) property conversion to short-term rental use increases vulnerability, requiring panel data tracking properties before and after conversion; (3) beachfront service location creates irreducible exposure that economic resources cannot mitigate; and (4) north-south geographic differentiation reflects persistent historical development patterns. These hypotheses require longitudinal data and causal research designs to move from pattern description to mechanism identification. Extension to Mediterranean islands, where sea-level rise projections threaten tourism infrastructure and cultural heritage across the basin ( Vandarakis et al. 2021), would test the transferability of the sectoral divergence documented here. Additionally, future work should investigate whether place attachment constrains adaptation even when economic resources are present ( Mortreux and Barnett 2017), a mechanism that would explain persistent hazard exposure in high-amenity coastal locations.

## 6. Conclusion

This study applied establishment-level risk indexing, fuzzy clustering, and exploratory spatial data analysis to 10,022 hospitality establishments across 400 km of Northern Adriatic coastline. The two sectors diverge sharply. Accommodation risk differentiates by hazard geography: converted residential properties concentrated in the Venice lagoon system score highest (HRI = 0.422), while purpose-built coastal hotels score lowest (HRI = 0.231). Service risk follows a simpler spatial logic, with beachfront establishments scoring highest (HRI = 0.440) regardless of economic positioning. This divergence maps onto a north-south geographic bifurcation, with northern Veneto concentrating converted residential accommodation in lagoonal hazard zones and southern Emilia-Romagna concentrating beachfront services along a linear, urbanized coastline. Services exhibit two- to four-fold stronger spatial autocorrelation than accommodations, consistent with agglomeration-driven clustering producing tighter spatial concentration than the amenity-driven sorting that characterizes accommodation location.

Component decomposition confirms that the accommodation structural pattern reflects genuine hazard geography rather than index construction circularity, while the market-based pattern is a vulnerability scoring artifact. External validation against Copernicus EMS flood delineations confirms that flood-proximate establishments score significantly higher in both sectors (p < 0.001), with the difference driven by the exposure component. This diagnostic approach, separating hazard-driven from vulnerability-driven associations at the component level, is transferable to composite index studies in comparable coastal settings. The open-source implementation, sensitivity-tested across 25 parameter combinations (CV = 9.4–10.1%), enables replication for other Mediterranean destinations where 77.3% of hospitality infrastructure concentrates within 1 km of the coastline.

The cross-sectional design constrains causal inference: observed HRI differences may reflect genuine vulnerability differences, selection effects, or confounding that longitudinal data would be needed to resolve. The divergent patterning between sectors and between northern and southern coastal zones identifies spatial variation that quasi-experimental designs can build upon, moving from the descriptive patterns documented here toward causal understanding. As adaptation costs rise disproportionately when the need for change becomes apparent, establishing where vulnerability concentrates is a necessary first step toward understanding why.

## Software availability

Source code available from: https://github.com/vgs549/HRI-NorthernAdriatic . Archived software available from: https://doi.org/10.5281/zenodo.19251594. License: [MIT]( https://opensource.org/licenses/MIT).

## Ethics and consent

Ethical approval and consent were not required for this study. The analysis uses publicly available spatial data on commercial hospitality establishments and does not involve human participants, personal data, or animal subjects.

## AI-Assisted tools disclosure

Generative AI assistance was used during the preparation of this manuscript. Specifically, Claude Code (Anthropic, v2.1.42, powered by Claude Opus 4.6) was used for: (1) generating Python scripts for figure creation (map visualizations and component plots); and (2) manuscript proofreading, including identification of citation inconsistencies, statistical verification against source data files, and editorial corrections. All AI-generated code was reviewed, tested, and validated by the author before use. All statistical claims were independently verified against analysis output files. The AI tool was not used to generate scientific content, interpret results, or draw conclusions. The author takes full responsibility for the content of this manuscript.

## Data Availability

The hospitality establishment database is described in Sales (2024). The dataset is deposited at Zenodo: https://doi.org/10.5281/zenodo.14929876 (Sales, V. G. 2024. “Dataset of Coastal Hospitality Establishments with Building Characteristics for the Northern Adriatic, Italy.” Zenodo. https://doi.org/10.5281/zenodo.14929876). Replication data for “Divergent risk patterns across coastal hospitality sectors in the Northern Adriatic” are deposited at Zenodo: https://doi.org/10.5281/zenodo.19134970. This deposit includes all values behind reported means, standard deviations, and other summary measures; the data underlying all figures and tables; extended data tables and figures; and variable descriptions for both accommodation and service sectors. Data are available under the terms of the Creative Commons Attribution 4.0 International license (CC-BY 4.0).
