# Supplementary material for: Divergent risk patterns across coastal hospitality sectors in the Northern Adriatic
Source: Open Res Eur. 2026 Jun 3;6:190. [Version 1] doi: 10.12688/openreseurope.23489.1 (PMC13550893; doi:10.12688/openreseurope.23489.1)
Supplement: Peer review history

# Reviewer report: Reviewer response for version 1

**DOI:** 10.21956/openreseurope.25427.r76159

**Published:** 2026-07-08

**Authors:** İlknur Zeydan

**Affiliations:** 1 Zonguldak Bülent Ecevit University, Zonguldak, Turkey

1) This study is a comprehensive and original work conducted in the North Adriatic coastal region.

 2) The sample is very well-chosen. A sample size of 10,022 is quite sufficient for the analyses.

 3) The literature review is well-conducted. The findings are well-supported by existing literature. Explanatory information is provided. The introduction, findings, and conclusion sections are comprehensively written.

 3) It is stated that accommodation and service facilities are located in a region that may be at risk of flooding. Therefore, adding information about what can be done in this regard to the conclusion section would improve the study.

 4) Adding a separate section at the end of the article, such as recommendations based on the findings, and suggesting solutions to the negative aspects identified would be a good improvement for the article. For example, what can the government do if some hotels and service facilities are in a flood zone?

If applicable, is the statistical analysis and its interpretation appropriate?

Yes

Is the study design appropriate and is the work technically sound?

Yes

Is the work clearly and accurately presented and does it engage with the current literature?

Yes

Are the conclusions drawn adequately supported by the results?

Yes

Are sufficient details of methods and analysis provided to allow replication by others?

Yes

Are all the source data and materials underlying the results available?

Yes

Reviewer Expertise:

Business administration, Marketing, Tourism, Sustainability

I confirm that I have read this submission and believe that I have an appropriate level of expertise to confirm that it is of an acceptable scientific standard.

# Reviewer report: Reviewer response for version 1

**DOI:** 10.21956/openreseurope.25427.r76160

**Published:** 2026-07-06

**Authors:** Anna Boqué-Ciurana

**Affiliations:** 1 Rovira i Virgili University, Tarragona, Catalonia, Spain

**Reviewer Comments:**

 This manuscript addresses a relevant and timely topic. The overall research approach is sound, and the conceptual framework based on the interaction between **hazard, exposure, and vulnerability** is appropriate and well aligned with current risk assessment frameworks. The study is generally well structured, the research questions are clearly presented, and the results provide valuable insights for tourism planning and climate adaptation.

 However, several aspects require further clarification and strengthening before indexing

 .

**General Comments:**

 One of the main strengths of the manuscript lies in the detailed treatment of the **exposure** and **vulnerability** dimensions. Both components are comprehensively developed and supported by relevant indicators. In contrast, the **hazard** dimension appears comparatively underdeveloped. The analysis focuses exclusively on **sea-level rise**, while other climate-related hazards that are highly relevant for coastal hospitality systems (e.g., coastal flooding, storm surges, marine heatwaves, extreme precipitation, droughts, or heat stress) are not considered.

 Although sea-level rise is undoubtedly an important hazard for coastal destinations, the manuscript should either justify more explicitly why it was selected as the sole hazard indicator or acknowledge this approach as an important limitation of the study.

 Furthermore, the sea-level rise projections appear to be based on relatively outdated climate scenarios. The use of **RCP 4.5 as the only emission scenario** raises some concerns, particularly given the widespread adoption of the more recent **Shared Socioeconomic Pathways (SSPs)** in contemporary climate impact assessments. At minimum, the authors should provide a stronger justification for relying exclusively on RCP 4.5. Ideally, the analysis should be expanded to include multiple climate scenarios in order to better capture uncertainty and improve the robustness of the results (or better justify why only they are using RCP 4.5).

**Specific Comments:**

**Abstract**

 The abstract is well written and clearly presents the research topic, study objectives, methodological approach, and main findings.

**Introduction**

 The introduction is generally well developed, and the research questions are clearly stated.

 However, the final paragraph of the introduction includes a summary of the main findings. In the reviewer's opinion, this section is not the appropriate place to present research results. The introduction should focus on establishing the context, rationale, objectives, and research questions, while the findings should be reserved for the Results and Discussion sections.

**Literature Review**

 The literature review is appropriate and adequately supports the research background.

**Methodology**

- Figure 1 should include a north arrow to facilitate geographical interpretation.
- The manuscript should explicitly acknowledge the use of RCP-based projections rather than SSP-based projections and discuss the implications of this choice as a methodological limitation.
- It is unclear whether sea-level rise is the only hazard variable included in the risk assessment. This should be clarified more explicitly in the methodology section.
- Given the complexity of the analytical framework, the inclusion of a methodological workflow diagram would significantly improve readability and reproducibility.
- A positive aspect of the methodology is the consideration of the combined effects of hazard, exposure, and vulnerability, which provides a more comprehensive assessment than approaches focusing on a single dimension.

**Results and Discussion:**

 The results are clearly presented. However, some of the main methodological limitations should be discussed more explicitly, particularly:

- The exclusive use of **RCP 4.5**.
- The absence of alternative climate scenarios.
- The reliance on **sea-level rise as the sole hazard indicator**.
- The use of older climate projection frameworks instead of the more recent SSP-based scenarios.

These limitations may influence the magnitude and direction of the projected risks and should therefore be clearly acknowledged in the Discussion and Limitations sections.

**Recommendations for Future Research**

 Future studies should consider:

- Incorporating additional climate hazards relevant to coastal hospitality systems.
- Using updated climate projections based on SSP scenarios.
- Conducting multi-scenario analyses to assess the sensitivity of results to different emission pathways.

**Overall Recommendation:**

 The manuscript addresses an important research topic and presents a solid conceptual framework. Nevertheless, the hazard component requires further development and justification, particularly regarding the exclusive use of sea-level rise and the reliance on a single RCP 4.5 scenario. Addressing these issues would substantially strengthen the scientific robustness and policy relevance of the study.

If applicable, is the statistical analysis and its interpretation appropriate?

I cannot comment. A qualified statistician is required.

Is the study design appropriate and is the work technically sound?

Partly

Is the work clearly and accurately presented and does it engage with the current literature?

Yes

Are the conclusions drawn adequately supported by the results?

Partly

Are sufficient details of methods and analysis provided to allow replication by others?

Partly

Are all the source data and materials underlying the results available?

Yes

Reviewer Expertise:

Climate Services, climatology, co-creation, tourism

I confirm that I have read this submission and believe that I have an appropriate level of expertise to confirm that it is of an acceptable scientific standard, however I have significant reservations, as outlined above.

# Reviewer report: Reviewer response for version 1

**DOI:** 10.21956/openreseurope.25427.r76164

**Published:** 2026-07-06

**Authors:** Diana de Yta-Castillo

**Affiliations:** 1 Autonomous National University of Mexico, Mexico City, Mexico City, Mexico

Congratulations to the author of the manuscript. It is a well-written article with solid and interesting arguments.

 I offer the following observations to take the opportunity to improve the article.

 It would be beneficial for the discussion to reflect on its contributions to public policy and to tourism sector managers.

 For example, it would be valuable for the discussion to address how authorities at all levels of government should support the tourism enterprises analyzed, in order to bolster their adaptive capacity in the face of the hydrometeorological events mentioned in the article.

 I also suggest not referring to disasters as "natural disasters," even though some academic literature continues to use that term; because, as the United Nations Office for Disaster Risk Reduction (2020) points out, disasters are the result of human actions and decisions.

If applicable, is the statistical analysis and its interpretation appropriate?

Yes

Is the study design appropriate and is the work technically sound?

Yes

Is the work clearly and accurately presented and does it engage with the current literature?

Yes

Are the conclusions drawn adequately supported by the results?

Yes

Are sufficient details of methods and analysis provided to allow replication by others?

Yes

Are all the source data and materials underlying the results available?

Yes

Reviewer Expertise:

Resilience and environmental management of tourism.

I confirm that I have read this submission and believe that I have an appropriate level of expertise to confirm that it is of an acceptable scientific standard.

# Reviewer report: Reviewer response for version 1

**DOI:** 10.21956/openreseurope.25427.r76165

**Published:** 2026-06-30

**Authors:** Enes Karadeniz

**Affiliations:** 1 Faculty of Arts and Sciences, Department of Geography, Inonu University, Malatya, Turkey

I have carefully reviewed the manuscript. The study compares the spatial risk patterns of accommodation and service establishments along the Northern Adriatic coast and develops a Hospitality Risk Index that combines the Hazard, Exposure, and Vulnerability components. The large establishment level dataset, fuzzy clustering, H3 spatial indexing, and open data approach are among the strengths of the study. However, important revisions are needed regarding the structure of the index, the validity of some proxy variables, spatial scale selection, the validation approach, and figure presentation.

1. The Abstract generally reflects the study well. However, expressions such as “demonstrates” and “validation confirms” should be written more cautiously, considering the small to moderate effect sizes.
2. The Introduction and Literature Review establish the research gap effectively. However, the location logics of the sectors and the expected risk differences are repeated several times, making the text unnecessarily long.
3. The four descriptive expectations resemble hypotheses that are effectively tested in the analyses. They should be linked more clearly to the research questions, or the authors should explain why they are not presented as hypotheses.
4. The use of IPCC AR5 RCP4.5 and 2030 projections in the Hazard component should be justified. The authors should explain why the current AR6 scenarios were not preferred and clarify the vertical datum compatibility with the elevation data.
5. The HRI formula and variable transformations are not sufficiently transparent in the main text. The component, direction, normalization, threshold, and weight of each variable should be presented in a summary table.
6. Price and user rating scores do not directly measure actual adaptive capacity. These variables should be defined as proxies for economic and operational positioning rather than insurance, preparedness, or post disaster recovery capacity.
7. Coding missing values as -1 in categorical variables may create artificial classes. In addition, the sample size decreases substantially in some clustering scenarios, particularly in the service amenity analysis, which includes only 9% of the total sample.
8. The main details of the Fuzzy C-means implementation should be summarized in the main text. The selection of the number of clusters, the fuzzifier parameter, variable standardization, the number of repetitions, and the calculation of the F1 score in unsupervised clustering should be explained.
9. H3 resolution 7 and the 25 km neighborhood threshold may affect the spatial results. A brief sensitivity analysis using alternative resolutions and distance thresholds should be provided.
10. There is partial overlap between the clustering variables and the HRI components. Although removing the Vulnerability component is a useful check, the remaining associations should not be interpreted as entirely independent evidence of spatial risk.
11. The comparison based on flood events is a valuable validation step. However, the 50 m buffer should be tested using alternative distances, and the riverine flooding component of the Emilia-Romagna event and the spatial dependence among observations should be considered.
12. It is not explained how multiple local tests were corrected in the LISA and Gi* analyses. The number of permutations, weight standardization, treatment of isolated cells, and FDR correction should be clearly reported.
13. The directly measured results and theoretical explanations should be more clearly separated in the Results and Discussion sections. Interpretations of adaptive capacity and development pathways based on price, building type, and coastal location should be presented as possible explanations.
14. The cartographic presentation of the figures should be substantially improved. The map panels are small, while the color scales occupy excessive space, making the map details difficult to read. The inconsistencies among the numbering, content, and captions of Figures 2-5 should also be checked throughout.

If applicable, is the statistical analysis and its interpretation appropriate?

Partly

Is the study design appropriate and is the work technically sound?

Partly

Is the work clearly and accurately presented and does it engage with the current literature?

Partly

Are the conclusions drawn adequately supported by the results?

Partly

Are sufficient details of methods and analysis provided to allow replication by others?

Yes

Are all the source data and materials underlying the results available?

Yes

Reviewer Expertise:

Environmental sciences, physical geography, GIS and spatial analysis, remote sensing, spatial statistics, land-use and land management, ecological modelling, environmental risk assessment, and spatial planning.

I confirm that I have read this submission and believe that I have an appropriate level of expertise to confirm that it is of an acceptable scientific standard, however I have significant reservations, as outlined above.
